# A novel low-cost electrode for recording the local field potential of freely moving rat’s brain

**DOI:** 10.1515/tnsci-2020-0104

**Published:** 2020-06-05

**Authors:** Xue-Feng Ding, Yan Gao, Hui Zhang, Yuan Zhang, Shao-Xia Wang, Yong-Qi Zhao, Yi-Zheng Wang, Ming Fan

**Affiliations:** Institute of Military Cognition and Brain Sciences, Beijing, P. R. China; Institute of Radiation Medicine, Beijing, P. R. China; Department of Neurosurgery, Air Force Medical Center of PLA, Beijing, P. R. China; Laboratory of Neural Circuit Plasticity, School of Brain and Cognitive Sciences, Beijing Normal University, Beijing, P. R. China

**Keywords:** beta oscillation, local field potential, Parkinson’s disease, recording electrode

## Abstract

Local field potentials (LFPs) are involved in almost all cognitive activities of animals. Several kinds of recording electrodes are used for recording LFPs in freely moving animals, including commercial and homemade electrodes. However, commercial recording electrodes are expensive, and their relatively fixed size often causes a steric hindrance effect, especially when combining deep brain stimulation (DBS) with LFP recording, which may not always satisfy the aim of researchers. Currently, an increasing number of researchers are designing their own recording electrodes to lower research costs. Nevertheless, there is no simple universal method to produce low-cost recording electrodes with a specific size according to the target brain area. Thus, we developed a simple method for quickly producing low-cost multiple-channel recording electrodes. To inspect the effectiveness of our self-designed electrode, LFPs were recorded in a Parkinson’s disease (PD) rat model, and an electrical stimulation electrode was implanted into the subthalamic nucleus to verify the space-saving ability of the self-designed recording electrode. The results showed that <30 min was needed to prepare an electrode and that the electrode materials cost <5 dollars. Further investigations showed that our electrode successfully recorded the beta oscillations (12–40 Hz) in the PD rat model. Thus, this method will greatly reduce the cost of recording electrodes and save time for researchers. Additionally, the small size of the electrode will further facilitate DBS research.

## Graphical Abstract

**Figure:**
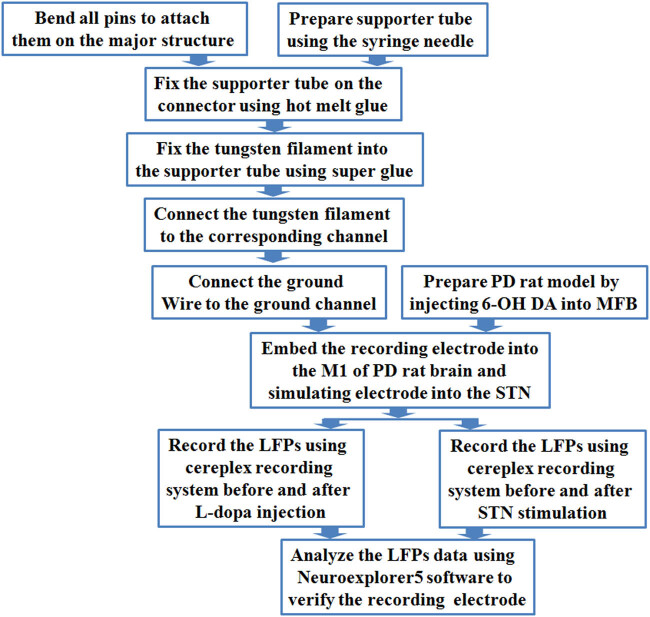

## 1 Introduction

Multiple channel electrodes work as a highly efficient tool to investigate the neuronal population activities, including local field potentials (LFPs) and single units, which greatly facilitate the neural circuit of brain diseases and animal behavior research [1,2,3]. The phases of oscillatory LFPs are involved in cognitive, perceptual, and motor processing. LFP recording and analysis has been widely used to characterize various brain disorders, such as Alzheimer’s disease (AD), schizophrenia, and Parkinson’s disease (PD) [4,5,6]. Among these brain disorders, numerous LFP data were recorded from the PD patient and animal model to decode its brain circuit. It has been found that the beta-band (12–40 Hz) oscillations are consistently strengthened with dopamine loss both in PD patients and parkinsonian animal models, while levodopa and deep brain stimulation (DBS) have been witnessed to improve PD symptoms and decrease the beta oscillations [7,8,9,10]. Thus, a good recording electrode will be a powerful tool to investigate LFPs and reveal the mechanisms underlying brain disorders. Several commercial recording electrodes are available and widely used all over the world, such as products from Plexon Inc, Blackrock Microsystems LLC, and Neuralynx Corp. A large number of reports have demonstrated their effectiveness in recording the LFP and spike firing in the brain [11,12,13,14,15]. However, all of them are very expensive, with a 16-channel implantable recording electrode costing at least approximately 100 dollars, which has undoubtedly greatly increased the cost of the research work. Particularly, the implantable recording electrode is usually one-off and thus will incur a high cost to finish a project concerning *in vivo* electrophysiological experiments. Moreover, the size of the commercial recording electrode is usually fixed, and researchers are unable to modify its structure by themselves, which limits its flexibility and may cause a steric hindrance effect in combination with other recording or stimulation electrodes, especially when performing DBS-related research, considering the limited surface area of the rodent brain. For the aforementioned reasons, many labs worldwide prepare the recording electrode by themselves to lower costs and reduce steric hindrance, which has also contributed to growing research on neuronal population activities [16,17,18,19,20]. Nevertheless, there is no universal and reproducible protocol for quickly producing such a low-cost recording electrode with a small size, which is especially important to save time and money. For this purpose, here, we utilize some typical low-cost materials in the neuroscience lab to prepare a recording electrode using a very simple procedure. Due to our extensive experience in recording beta oscillation in the PD rat model, we chose the PD rat model to verify the effectiveness of our self-designed recording electrode [21,22,23,24]. We hope that this simple and reproducible method is effective in quickly preparing electrodes to record LFPs of freely moving animals and can overcome the aforementioned limitations of the commercial recording electrode to further facilitate LFP-related behavioral research.

## 2 Materials and methods

**Figure 1:**
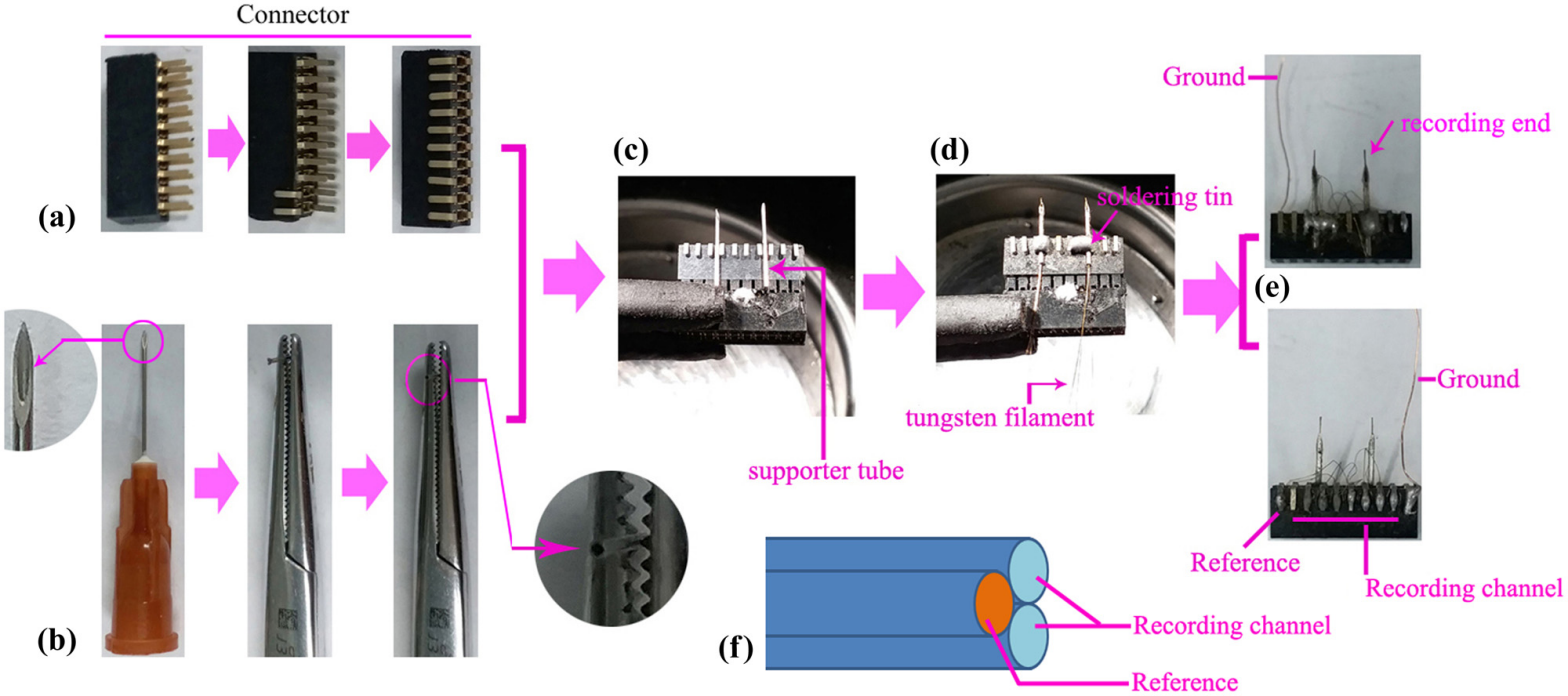
The procedure for producing the recording electrode (a bilateral recording electrode is shown as an example). (a) The procedure for preparing the framework structure of the bilateral recording electrode. A 20 pin connector (2 × 10) was used as a framework material, and each pin of the connector was bent to attach it to the major structure of the connector. (b) The procedure for preparing the supporter tube. The syringe needle (diameter = 0.45 mm) of a 1 mL injection syringe was cut with pliers and fixed with forceps, and the blunt end was polished with a knife grinder to expose the cavity. (c and d) The method for connecting the supporter tube and framework structure. The connector was fixed horizontally, and the two supporter tubes were placed on the framework structure and fixed with a soldering tin or a transparent rubber. The distance between the two tubes was determined according to the distance of the two target brain regions. Next, the tungsten filament was placed in the supporter tubes, and super glue was slowly and carefully added to the tip of the supporter tube to fix the tungsten filament. (e) The recording end was cut using sharp eye scissors, and the other end was connected to the reference (R) channel and recording channels. A copper wire (length = 2–3 cm) was connected to the ground (G) channel for use as the ground electrode. (f) A schematic diagram of the recording end. One was used as a reference line, and the others were used for recording.

A connector (20 pin, 2 × 10) was used as the framework structure (as shown in Figure 1a). As shown in Figure 1b, the syringe needle (diameter = 0.45 mm) of a 1 mL injection syringe was cut with pliers and fixed with forceps, and the blunt end was polished with a knife grinder to expose the cavity, which was used as the supporter tube of the tungsten filament. Soldering tin or hot melt glue was used to fix the supporter tubes on the fixed framework structure. The tungsten filament (diameter = 0.1 mm, Figure 1d) was used to record the filament and reference. A copper wire (length = 2–5 cm, diameter = 0.2 mm) was used as ground (G) wire.

### 2.1 The design of a recording electrode

As shown in Figure 1a, a 20 pin connector (2 × 10) was used as a framework structure. Each pin of the connector was bent to attach it to the major structure of the connector. As shown in Figure 1b, the syringe needle (diameter = 0.45 mm) of a 1 mL injection syringe was cut with pliers and fixed with forceps, and the blunt end was polished with a knife grinder to expose the cavity, which was used as the supporter tube of the tungsten filament. Next, the connector was fixed horizontally, and the supporter tubes were fixed on the connector using soldering tin or hot melt glue (Figure 1c and supplement Figure 1b). The number of supporter tubes was determined according to the number of target areas, and the distance between the two tubes was determined according to the distance of the two target brain regions. However, we found that the hot melt glue was better than soldering tin in fixing the supporter tube, which also saved additional space (Supplemental Figure 1). Next, the tungsten filament was placed in the supporter tubes, and super glue was slowly and nominally added to the tip of the supporter tube to fix the tungsten filament (diameter = 100 µm, Figure 1d). According to the purpose of the research, the number of tungsten filaments in each supporter tube should range from 2 to 5 (the size of the supporter tube can accommodate five tungsten filaments at most); one of the tungsten filaments was used as a reference, and the others were used as recording channels. Thus, each supporter tube would be a single-channel electrode (two tungsten filaments, one as a reference electrode and the other as a recording electrode) or multiple-channel electrode (3–5 tungsten filaments) according to the number of tungsten filaments. After the super glue was fully set, the recording end of the tungsten filament was quickly cut with eye scissors to expose the tungsten filament core. The other end of the tungsten filament was cut to a proper length, the insulating layer was removed with a subtle tweezer, and it was connected to the reference (R) and recording channels, respectively. A copper wire (diameter = 200 µm, length = 2–3 cm) was connected to the ground (G) channel for use as the ground wire (Figure 1e).

### 2.2 Defining the channel map of the recording electrode

**Figure 2:**
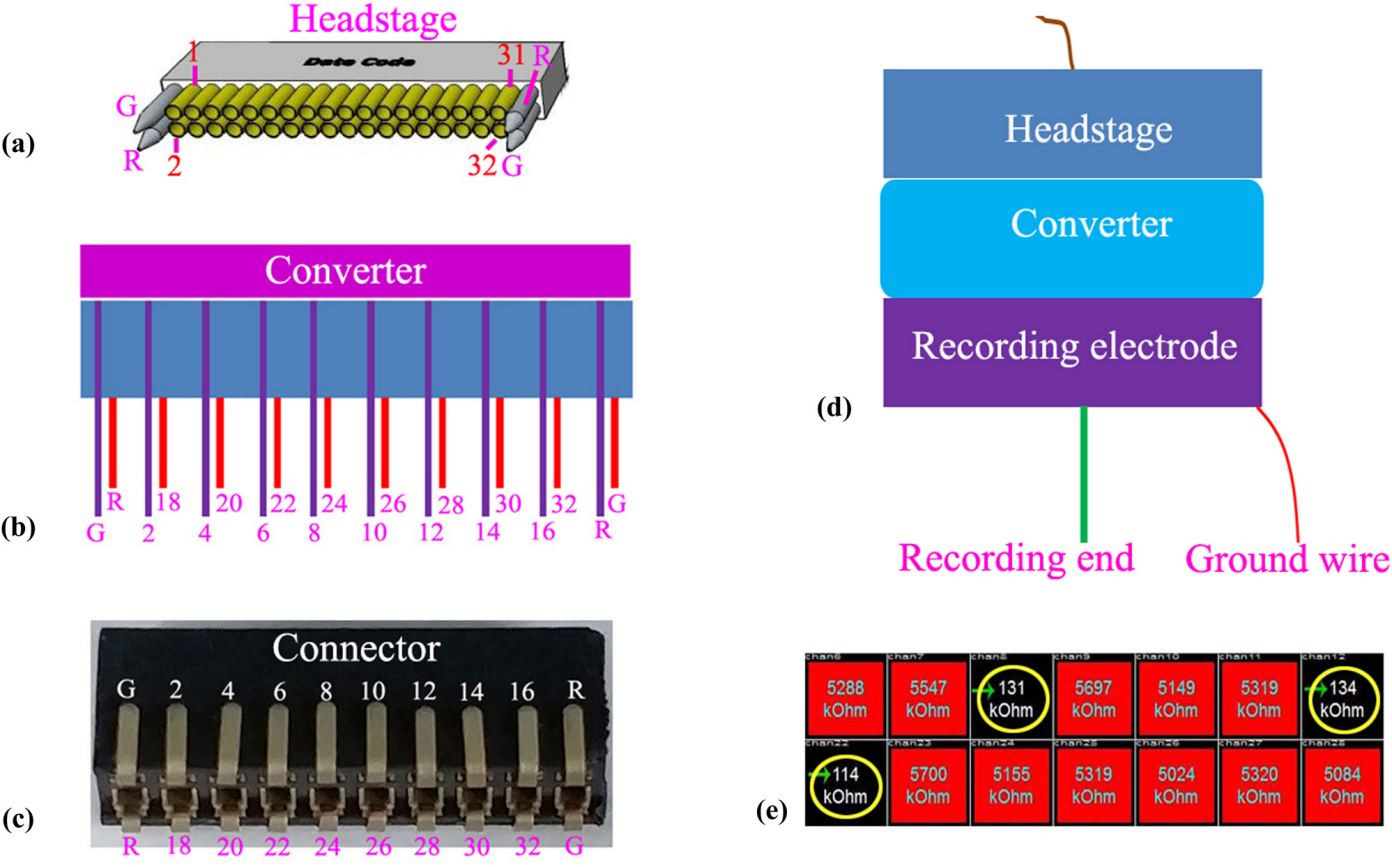
Definition of the recording channels. (a) The channel map of the headstage. (b) The channel map of the converter. (c) The channel map of the recording electrode. (d) Schematic diagram of the combinatory headstage, converter, and recording electrode. (e) Impedance of the self-designed recording electrode.

As shown in Figure 2a, the definition of the channel was determined by the headstage of Blackrock Microsystems (Blackrock Microsystems LLC, Salt Lake City, USA). To adapt our designed bilateral electrode, a converter (shown in Figure 2b) was created according to the channel definition of headstage; specifically, the even channels (2, 4, 6,…,32) of the headstage were connected to the corresponding channels (2, 4, 6,…,32) of the converter. Thus, the channel definition of the recording electrode was determined as even channels (2, 4, 6,…,32). As shown in Figure 2d, the converter was used to connect the headstage to the recording electrode when recording the LFPs and single units. Additionally, we detected the impedance of the electrode. As shown in Figure 2e, the impedance value of a three-channel recording electrode with one recording end was examined, and the impedance of each channel was 131 kOhm, 134 kOhm, and 114 kOhm, respectively, which is appropriate for recording LFPs.

### 2.3 Animals

Male Sprague Dawley (SD) rats weighing 200 ± 10 g were used in this study (all the rats were purchased from Beijing Vital River Laboratory Animal Technology Co., Ltd, Beijing, China). The animals were housed up to two rats per cage with a 12-hour light/dark cycle at constant temperature and humidity, and food and water were freely available.


**Ethical approval**: The research related to animal use has been complied with all the relevant national regulations and the guidelines on animal welfare of the Institute of Military Cognition and Brain Sciences, and all efforts were made to ameliorate animal suffering.

### 2.4 Preparation of the PD rat model

The hemi-parkinsonian rat model was prepared by injecting 2,4,5-trihydroxyphenylalanine (6-OH DA; Sigma, USA; total volume = 3 µL, 5 µg/µL) into the right medial forebrain bundle (4.3 mm posterior and 1.5 mm lateral to the bregma, 7.6 mm depth from the skull surface) of SD rats (200 g ± 10 g). After injection, the needle was left in place in the target for 5 min to allow the drug solution to diffuse completely. Two weeks after injection, apomorphine (1 mg/kg body weight; Sigma, USA) was intraperitoneally injected to induce contralateral rotations to evaluate establishment of the model. The lesion was considered successful in those animals that made ≥15 net counterclockwise rotations in 3 min [24,25]. Madopar (purchased from Roche [40 mg levodopa + 12.5 mg benserazide]/kg body weight) was injected into the enterocoelia of the PD rat to eliminate the beta oscillations.

### 2.5 Surgery and electrode placement

Male SD rats were fully anesthetized and then fixed in a stereotaxic frame. The skull was exposed, burr holes were created above the electrode placement using a micro bone drill, and a small amount of dura mater was removed. Bone nails (5–6 μ) were fixed at the none-target site on the skull to further fix the electrode with dental cement. Next, the recording electrode was placed in the target brain areas with stereotaxic micromanipulators (David Kopf Instruments, Tujunga, CA, USA) and then fixed with dental cement. To prevent the holes from stimulating the electrode, subthalamic nucleus (STN) was blocked with dental cement in advance using a bone nail. Subsequently, the recording electrode was fixed, and the stimulating electrode was placed at STN and fixed with dental cement. In this study, both sides of M1 (2.5 mm anterior and 2.5 mm lateral to the bregma, at a depth of 2 mm from the skull surface) were target area of the recording electrode; a stimulating electrode was implanted at the STN, and the ground filament was inserted randomly in a non-target region [26,27].

### 2.6 Recording and stimulating parameters and signal processing

LFPs were recorded using a Blackrock detecting system (Blackrock Microsystems LLC, Salt Lake City, USA), and continuous data were analyzed using NeuroExplorer 5.0 software (NeuroExplorer, Colorado Springs, USA). The spectrum and power spectral density of continuous data were calculated. The LFP was extracted from the raw broadband signal recorded in the M1 zone by bandpass filtering between 1 and 250 Hz. Band-limited LFP signals were generated with the following bandwidth settings: beta (12–40 Hz) [26,28]. A constant current-isolated stimulator (Digitimer, Welwyn Garden City, Hertfordshire, UK) delivered continuous electrical pulses to the STN electrodes at an intensity below the threshold for induced movement (50–250 mA) [29].

## 3 Results

### 3.1 The self-designed recording electrode is low cost and easy to prepare

As described in Section 2, the procedure is simple and easy to master (as shown in Figure 1 and the graphical abstract), and the preparation of such a recording electrode requires <30 min, saving large amounts of time for researchers. Moreover, all the materials used for our electrode are common and cheap, and they can easily be purchased from the electrical market. As shown in Table 1, a recording electrode costs <5 dollars, which is much cheaper than the commercial recording electrode (approximately 100 dollars each), greatly reducing the costs for researchers.

**Table 1:** The cost of the materials in preparing the recording electrode

Item	Quantity	Unit price ($)	Total ($)
Connect or (2 × 10 pin)	1	<0.7	<0.7
Syringe needle	1	<0.1	<0.5
Tungsten filament (*d* = 0.1 mm)	1 cm	<0.2	<2.0
Polyurethane rubber	1 cm^3^	<0.2	<0.4
Copper wire (*d* = 0.2 mm)	1 cm	<0.1	<0.6
Super glue	50 µL	<0.2	<0.4
Soldering tin		<0.2	<0.4
Total ($)			<5.0

*d*: diameter

### 3.2 The self-designed recording electrode successfully acquires LFP data from freely moving rat’s brain

**Figure 3:**
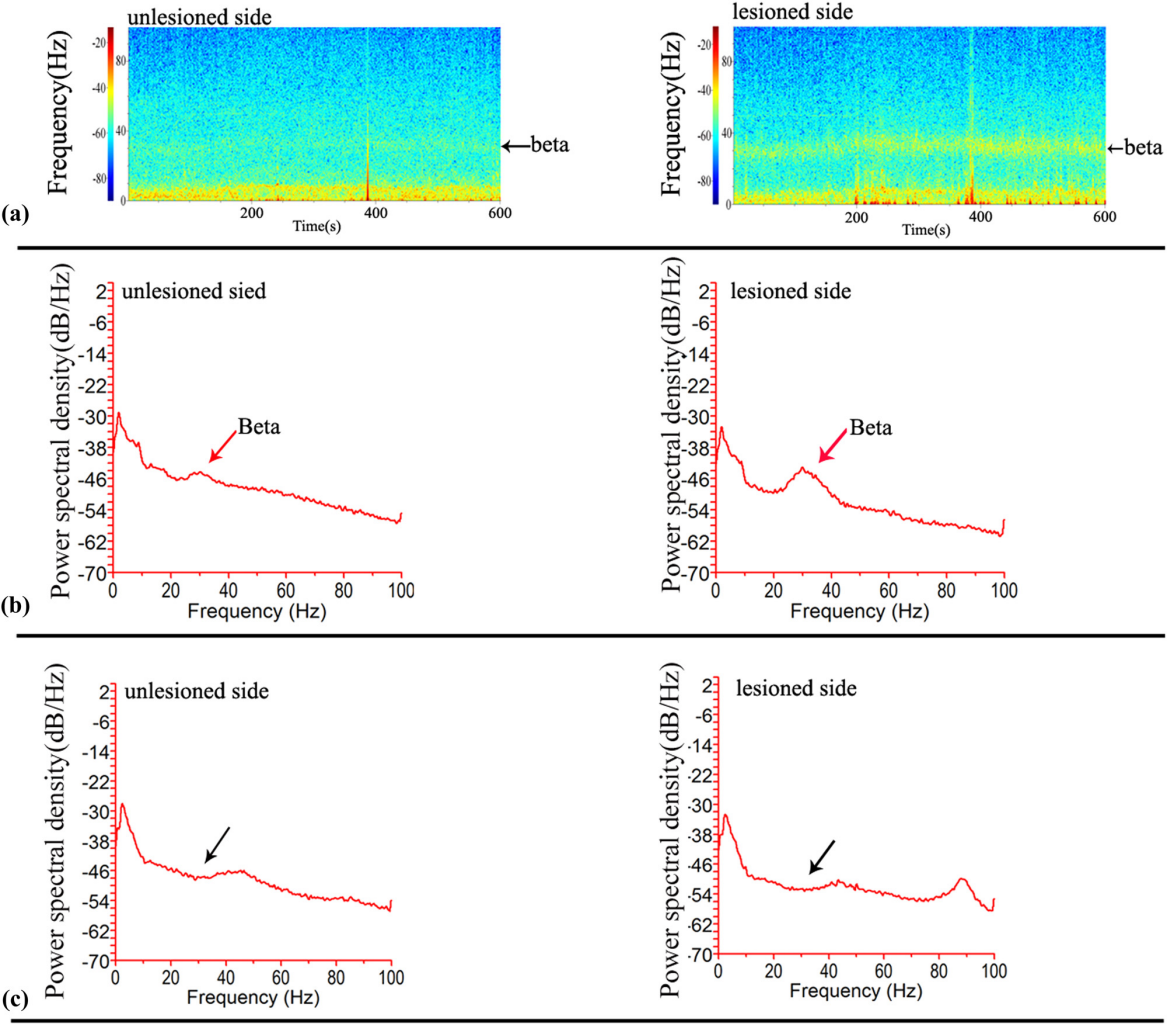
The self-designed electrode successfully acquired LFPs in the freely moving PD rat model. (a) The spectrum analysis of continuous data showed that the beta band was found approximately 31 Hz on both the sides of M1, and the intensity of the beta band was much weaker on the non-lesion side than the lesion side. (b) Power spectral density analysis showed that the peak frequency value of beta oscillations was found to be approximately 31 Hz. (c) Madopar remarkably decreased the peak of the beta band 40 min after injection.

Beta oscillations (12–40 Hz) were usually found in the primary motor cortex (M1 zone) of the hemi-parkinsonian rat induced by 6-OH DA [26,28,29]. Thus, to test our self-designed recording electrode, LFPs in the M1 zone of the cortex in the freely moving PD rat model were examined using our designed bilateral electrode combined with the Blackrock detection system (Blackrock Microsystems LLC, Salt Lake City, USA). Ten minutes of continuous data at a time were recorded for 100 min. The Cereplex system showed that the continuous data were successfully recorded. As shown in Figure 3, by analyzing the continuous data using NeuroExplorer 5.0 software (NeuroExplorer, Colorado Springs, USA), including the spectrum and power spectral density of continuous data, we found that our designed electrode successfully recorded the beta oscillation on both sides of the M1 cortex and the beta band was located approximately 30 Hz, though the beta oscillation on the lesion side was significantly stronger than on the non-lesion side (Figure 3a). Further analysis of the power spectral density showed that the peak frequency of beta oscillation was located at 30 Hz, which is consistent with the results of the spectrum calculations [7]. To further confirm the beta oscillations, l-dopa, which is widely used in PD patient therapy and therapeutic studies in the PD rat model, was injected into the abdomen in the PD rat model (40 mg/kg body weight), and continuous data were recorded 40 min after injection. As shown in Figure 3c, the beta peak was removed, which demonstrated that the beta oscillation was eliminated by l-dopa, which is consistent with previous work [26,30,31].

### 3.3 The self-designed electrode displays a good space-saving effect when combined with STN stimulation

**Figure 4:**
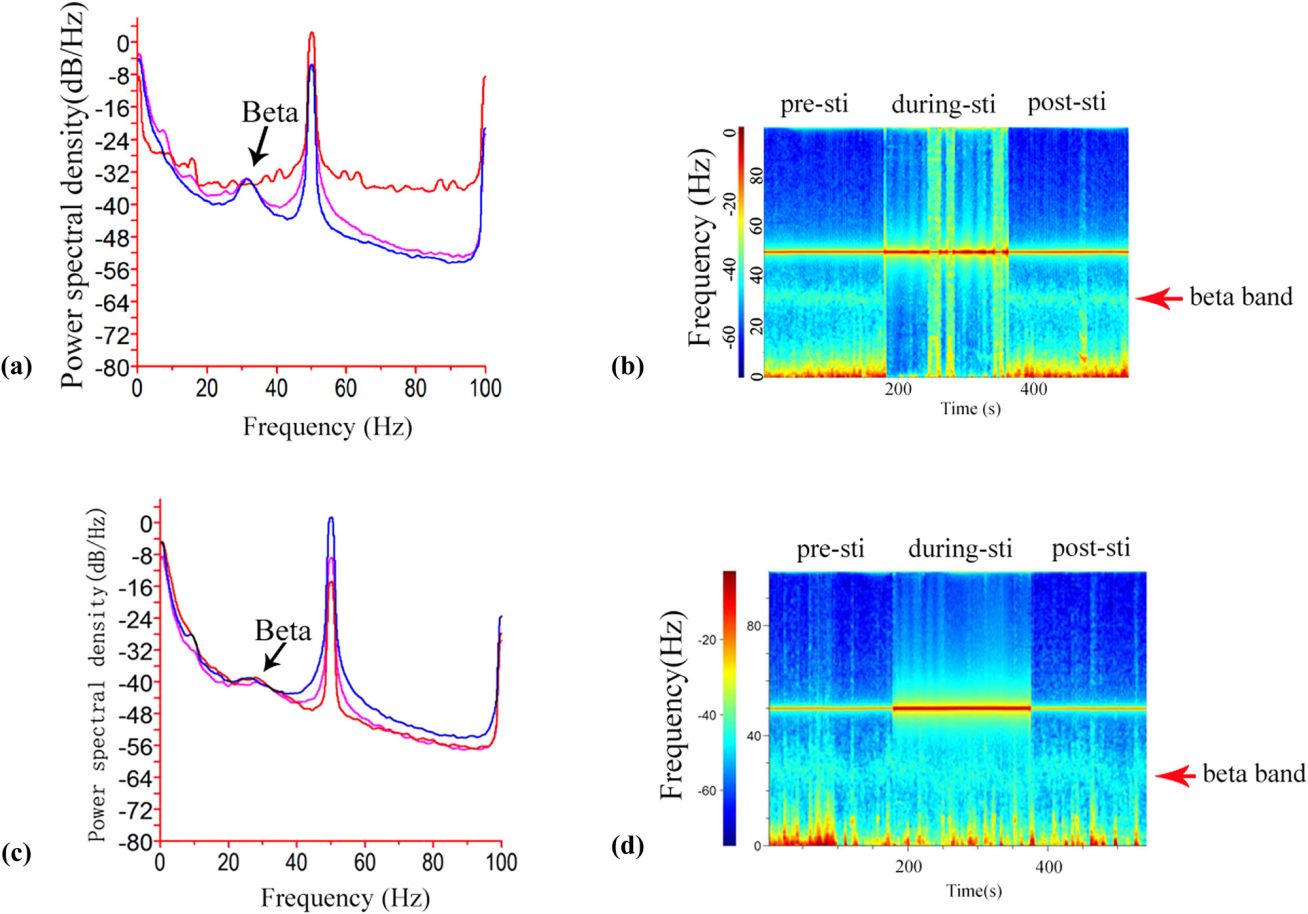
The self-designed recording electrode displays good space saving ability combined with STN stimulation. (a) Power spectral density analysis of LFPs acquired before stimulation, during stimulation, and after stimulation using 100 Hz electric stimulation. The beta oscillation band (31 Hz) was eliminated by the 100 Hz electric stimulation. (b) Spectrum analysis of LFPs before stimulation, during stimulation, and after stimulation using 10 Hz electric stimulation. The 10 Hz electric stimulation could not remove the beta band. (c and d) Power spectral density spectrum analysis of LFPs acquired before stimulation, during stimulation, and after stimulation using 10 Hz electric stimulation.

A paradox between the relatively large size of the recording electrode and limited area of the rodent brain is consistently observed in most neuroscience labs. The smaller the size of the electrode, the less the steric hindrance produced, when recording more than two target areas close together. Due to previous reports showing that high-frequency STN electric stimulation can remove the beta oscillation in the M1 cortex in the PD rat model [7,29,32,33], to test the effectiveness of our designed recording electrode in reducing steric hindrance, we implanted our self-designed recording electrode in the M1 cortex and a stimulating electrode in the STN (the distance between M1 and STN was 5 mm in our experiment). The results showed that at least 3 mm is present between the recording electrode and stimulating electrode, and the LFP data for M1 were successfully acquired before electrical stimulation (3 min, pre-sti), during stimulation (3 min, during-sti), and after stimulation (3 min, post-sti) continuously. As shown in Figure 4a, power spectral density analysis showed that 100 Hz STN stimulation successfully removed the beta oscillations in the PD rat model. The spectrum analysis also demonstrated the same result (Figure 4b), which is consistent with previous reports. In contrast, both power spectral density analysis and spectrum analysis showed that 10 Hz STN stimulation failed to remove the beta oscillations in the PD rat model [29]. These results showed that our self-designed recording electrode displayed good space saving specialty, which also confirmed the beta oscillations found before using our self-designed recording electrode.

## 4 Discussion

LFPs are generated by the extra-cellular electric fields that are induced by neural activity, mainly by transmembrane currents. The phases of oscillatory LFPs were involved in cognitive, perceptual, and motor processing [34,35,36,37]. To further understand how neural oscillations contribute to cognition, it is crucial to understand how LFP phases are related to those of the underlying neural activity. Thus, to capture the LFP signal, the implantable recording electrode for capturing LFPs from freely moving animals plays a crucial role. Currently, increasing amounts of LFP data are obtained from patients with various brain disorders and in related animal models, such as AD and PD. Among these diseases, LFP recording and analysis play a key role in decoding PD, and levodopa and DBS have been widely used to modulate beta oscillations in PD patients and to treat PD symptoms [7,8,9,10]. However, commercial implantable recording electrodes are expensive and one-off in recording LFPs in freely moving animals, forcing more and more neuroscientists to prepare an implantable electrode by themselves. Nevertheless, there is no universal efficient method to prepare low-cost recording electrodes. In this study, we developed a simple method to design and prepare an implantable electrode with a small size. The specific procedure is shown in Figure 1, and it is very simple such that even a beginner can grasp it easily. Additionally, according to our experiences, preparing a recording electrode requires <30 min, saving time for researchers. Moreover, all the materials used in the electrode are common and rather low cost (Table 1), so they can be acquired easily, which will undoubtedly save large amounts of money for researchers. Because only 16 channels can be used for recording in our self-designed electrode, there were 32 channels or more in the commercial headstage in our detection system. Thus, we needed to design a converter to connect the headstage and recording electrode when capturing LFP signals. As shown in Figure 2, the even channels (2,4,6,…,32) of the headstage were connected to the relevant channels of the recording electrode with the converter. Though only 16 channels at most can be used in our self-designed electrode, this is sufficient to simultaneously capture the LFP signals from two or more areas of the animal brain. Researchers can design two or more recording ends in the recording electrode according to their needs. In addition, the impedance of each channel is approximately 120 kOhm, which is sufficient to capture the LFPs. Because beta oscillations are present in the M1 cortex of PD patients, the PD monkey model, and the PD rat model [38,39,40,41], we wanted to test our self-designed recording electrode by capturing beta oscillations in a freely moving PD rat model. Analysis of the unilateral lesion PD rat model showed that our self-designed recording electrode successfully acquired the beta oscillations from M1 in a freely moving PD rat model, confirming the effectiveness of the self-designed electrode (Figure 3). Furthermore, using our homemade recording electrode, we also successfully observed spike firing at the anterovent thalamic nucleus in the PD rat model (Supplemental Figure 2). To test the capacity to save space when combined with other electrodes, we simultaneously implanted our self-designed recording electrode targeting the M1 zone and a stimulating electrode targeting STN, and we found a distance of at least 3 mm between the recording and stimulating electrodes, which demonstrated that the self-designed recording electrode was able to save sufficient space for performing DBS at the other target area close to the recording electrode. LFP analysis showed that 100 Hz rather than 10 Hz STN stimulation could remove the beta oscillations, which also confirmed that our self-designed electrode could successfully acquire LFP signals from freely moving animals [29,32,33,34].

In summary, this study has outlined the design and verification of a simple and cheap electrode capable of recording multichannel LFP signals from freely moving rodents. The materials used in the electrode are common and cheap, and the electrode preparation procedure is simple and can be easily grasped even by beginners, which will greatly lower research costs, including money and time. The small size of the electrode makes it easy to implant. We believe that this method will further facilitate LFP-related research and decode the basis of animal behavior and neural circuits.
